# Cross-Detector Visual Localization with Coplanarity Constraints for Indoor Environments

**DOI:** 10.3390/s25247593

**Published:** 2025-12-15

**Authors:** Jose-Luis Matez-Bandera, Alberto Jaenal, Clara Gomez, Alejandra C. Hernandez, Javier Monroy, José Araújo, Javier Gonzalez-Jimenez

**Affiliations:** 1 Machine Perception and Intelligent Robotics Group (MAPIR-UMA), Malaga Institute for Mechatronics Engineering and Cyber-Physical Systems (IMECH.UMA), University of Malaga, 29071 Malaga, Spain; josematez@uma.es (J.-L.M.-B.); jgmonroy@uma.es (J.M.); 2 Ericsson Research, 16483 Stockholm, Sweden; ajg@uma.es (A.J.); clara.gomez@csic.es (C.G.); alejandra.hernandez.silva@ericsson.com (A.C.H.); jose.araujo@ericsson.com (J.A.)

**Keywords:** visual localization, image keypoints, long-term interaction, mobile robots

## Abstract

Most visual localization (VL) methods typically assume that keypoints in the query image are detected with the same algorithm as those stored in the reference map. This poses a serious limitation, as new and better detectors may progressively appear, and we would like to ensure the interoperability and coexistence of cameras with heterogeneous detectors in a single map representation. While rebuilding the map with new detectors might seem a solution, it is often impractical, as original images may be unavailable or restricted due to data privacy constraints. In this paper, we address this challenge with two main contributions. First, we introduce and formalize the problem of cross-detector VL, in which the inherent spatial discrepancies between keypoints from different detectors hinder the process of establishing correct correspondences when relying strictly on the similarity of descriptors for matching. Second, we propose CoplaMatch, the first approach to solve this problem by relaxing strict descriptor similarity and imposing geometric coplanarity constraints. The latter is achieved by leveraging 2D homographies between groups of query and map keypoints. This process involves segmenting planar patches, which is performed offline once for the map, and also in the query image, which adds an extra computational overhead to the VL process, although we demonstrated in our experiments that this does not hinder the online applicability. We extensively validate our proposal through experiments in indoor environments using real-world datasets, demonstrating its effectiveness against two state-of-the-art methods by enabling accurate localization in cross-detector scenarios. Additionally, our work validates the feasibility of cross-detector VL and opens a new direction for the long-term usability of feature-based maps.

## 1. Introduction

In this work, we consider the problem of visual localization (VL) [1], in which the pose of a camera is estimated from a query image, given a previously built map of 3D points. This task heavily relies on establishing reliable correspondences between the keypoints observed in the query image and the projections of the map points. The discrepancy or error for each correspondence pair serves to construct the cost function to be minimized, which results in the estimated camera pose. The fundamental assumption that supports this process is that each pair of matched points represents the same physical entity in the scene [2]. Crucially, standard VL pipelines assume that the keypoint detector used on the query image is identical to the one applied when the map was built. Clearly, this is not guaranteed in heterogeneous sensor setups, where the keypoint detector used on the query image differs from the one applied when the map was built.

To date, this situation, which we coin *cross-detector visual localization*, has not been addressed in the literature yet, despite its practical importance. The motivation for tackling this problem is driven by the constant emergence of new and better keypoint detectors, and it would be of great interest to reuse an existing visual map for any kind of feature. This is particularly relevant for heterogeneous robotic fleets, for which legacy robots and newer models with different sensors must share a common map. Furthermore, in the context of long-term SLAM, ensuring cross-detector interoperability allows for hardware upgrades without the computationally expensive need to re-map entire environments, preserving the utility of historical map data. At first glance, rebuilding the map with new detectors could be seen as a solution, but in many cases, this is impractical since the original images may no longer be available or, simply due to data privacy constraints, may prevent devices from sharing images, leaving only the map features accessible. Addressing this crucial research gap, *cross-detector VL* would avoid the need for rebuilding and maintaining a separate world representation for each keypoint detector.

Although, to the best of the authors’ knowledge, this problem has not been explored yet, it maintains parallelism with the translation of descriptors from different algorithms into a common representation where they can be compared. This is known as the cross-descriptor problem, which has recently been addressed by [3]. Yet, it is important to stress that, for this approach, it was assumed that the features were identified using the same detector, focusing only on translating descriptors.

**Figure 1:**
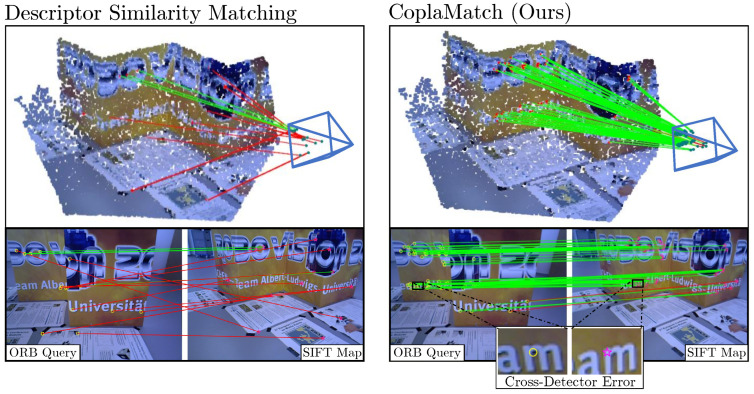
Example scenario of a camera using a corner detector (ORB) and trying to localize itself against a map built with SIFT keypoints (i.e., blobs). Note that, in order to ensure the comparability of both sets of keypoints, the same descriptor (i.e., BRIEF) was employed. Additionally, a map image is also depicted to illustrate the concept of the cross-detector error.

Figure 1 depicts an illustrative example in which a camera using a corner detector (concretely, ORB) attempts to localize itself against an existing map built using a SIFT detector (blob-based). Note that, to ensure that these keypoints are comparable, we employed the same feature descriptor, BRIEF, for both the query image and the map. As is evident from the top scenario in Figure 1, relying solely on descriptor information for feature matching results in sparse matches, with the majority being incorrect. We can also observe in the figure that most keypoints represent different physical entities that are spatially close in 3D space (see the zoomed-in area in the bottom-right corner). Intuitively, these keypoints could be used to establish correspondences between keypoints of a different nature if some extra information were provided to ease the matching.

This paper addresses this identified research gap through two contributions: first, we analyze whether minimizing reprojection errors is suitable for the cross-detector scenario, something that is not intuitive at first. Once validated, we introduce and formalize cross-detector VL, in which the inherent spatial discrepancies of keypoints representing not the same but close physical entities diminish the distinctiveness of descriptor-based correspondences and hinder feature matching. Secondly, to tackle this problem, we propose CoplaMatch, a novel method that establishes additional coplanarity constraints to establish more suitable correspondences between keypoints extracted with different detectors. Unlike traditional descriptor-based matching or recent learning-based methods such as SuperGlue [4], which struggle with fundamental differences in keypoint types or require specific retraining for each detector pair, CoplaMatch provides a detector-agnostic solution. This is achieved by leveraging homography transformations between keypoints from the map and the query image to impose coplanarity constraints. Unlike standard geometric methods (e.g., epipolar constraints [5]), which rely on verifying the identity of specific points across views, our approach uses set-to-set geometric verification. This aids in identifying correspondences among cross-detected keypoints that may not represent the exact same 3D point but are close enough (refer to Figure 1).

To enforce these constraints, keypoints must be annotated beforehand with their coplanar group indices, indicating which groups of keypoints are locally coplanar (i.e., within the query image or the map). This annotation process is conducted offline for the map and online for each query image, incurring a specific computational overhead that is later analyzed in detail. However, it is important to note that a limitation of this approach is its reliance on the presence of structural planes (e.g., walls), which restricts the method’s applicability to indoor environments or structured urban scenes.

Our approach is validated through extensive experiments using real-world data, for which different keypoint detectors are set for the map and the query image. However, to ensure comparability between their descriptors, for each experiment, the same feature descriptor is applied to both the map and the query image. Particularly to the evaluation of CoplaMatch, we only considered keypoints lying on structural planes in indoor environments (i.e., walls and planar objects attached to them such as pictures). The choice of structural planes is motivated not only because it reduces computational demands but also because structural planes are known for capturing environmental features that do not often change over time, thus facing long-term challenges [6]. The results demonstrate that CoplaMatch achieves precise localization results in this scenario. Although it incurs an extra computational overhead to apply the coplanar constraints for guiding the feature matching, it does not preclude its use for online applications (∼10–25 Hz, depending on the choice of the plane segmentation technique). Note that, while our experiments focus on indoor environments, our proposal could also be applied to outdoors with similar settings, such as urban areas with visible facades. In summary, our work provides the following contributions:

1. Formalizing the novel problem of cross-detector visual localization and analyzing its key challenge, the cross-detector error.
2. Proposing CoplaMatch, the first approach to solve cross-detector VL by leveraging geometric coplanarity constraints over descriptor similarity.
3. Providing an open-source implementation of our method, publicly available at https://github.com/EricssonResearch/copla-match (accessed on 11 November 2025).

## 2. Related Work

This section covers three different aspects related to the proposal in this work. First, it reviews methods for keypoint detection to then elaborate on current approaches to performing feature matching. Finally, a review of computer vision works employing coplanar constraints is presented.

### 2.1. Local Keypoint Detectors

Traditionally, local keypoint extraction from images has been performed through handcrafted algorithms, which are designed to detect certain types of interest points (e.g., corners, blobs, and ridges) that are repeatable and robust to changes (e.g., lighting conditions or different viewpoints). One of the widely adopted ones is the Scale-Invariant Feature Transform (SIFT) [7], which computes the Difference of Gaussians to detect blobs. Later, FAST [8] was presented as the first high-speed method for corner detection based on machine learning techniques. FAST was extended via ORB [9], which modifies the original algorithm to provide rotation invariance using the keypoint orientation. BRISK [10] was proposed as a binary corner detector built upon FAST but adding a scale-space to develop scale invariance.

Given the superior performance of neural network-based methods for many computer vision tasks, multiple learning-based keypoint detectors have been proposed. LIFT [11] is one of the first of these approaches. It is inspired by the traditional SIFT and demonstrates competitive results against handcrafted detectors. Similarly, MagicPoint [12] is a corner detection neural network that presented domain adaptation difficulties that its successor, SuperPoint [13], overcame, introducing homographic adaptation to boost performance. More recently, DeDoDe [2] introduced a novel approach by decoupling the detection and description processes, learning keypoints from 3D consistency. It should be noted that the choice of the keypoint detection algorithm depends on the application and its requirements, since each detector operates at a different rate, based on its computational complexity.

### 2.2. Local Feature Matching

Given two sets of local features, data association is typically performed under the assumption that corresponding keypoints represent the same physical entity in the scene. On this premise, the classical setting employs a nearest neighbor (NN) search to extract matches that are later filtered using techniques such as Lowe’s ratio test [7], cross-checking, and other heuristics [14,15]. Recently, leveraging the potential of neural networks, multiple learning-based methods have been proposed to improve feature matching. For instance, SuperGlue [4] relies on graph neural networks and attention mechanisms to learn priors about scene geometry and feature assignments, showing excellent performance but being hard to train. LightGlue [16] enhances the latter by reducing the training complexity and improving its performance. Another remarkable work is LoFTR [17], a detector-free approach that performs dense matching, which demonstrates impressive performance even in low-textured regions but at the cost of greater computational complexity. Building upon advancements in dense matching, RoMa [18] further improves robustness by combining strong pre-trained features from foundation models with a novel transformer-based decoder for robust dense feature matching. In the field of VL, [19] addresses VL through a learnable pipeline that relies on geometric descriptors instead of the traditional visual descriptors, reducing the storage requirements while relieving privacy concerns. Further addressing the challenges of VL, particularly in dynamic scenarios with significant appearance variations, other relevant works include InLoc [20], which leverages dense feature matching and view synthesis for robust large-scale indoor localization. Similarly, for long-term scenarios, methods like [6] employ retrieval-based strategies and robust correspondence verification to handle scene changes.

However, to the best of our knowledge, the crucial problem of cross-detector matching has not been previously addressed. This scenario fundamentally breaks the common assumption that keypoints from different images are extracted using the same detector. Consequently, traditional methods relying on descriptor similarity become unreliable or are entirely precluded. Furthermore, existing learning-based matching approaches are typically tailored to specific feature types, which inherently limits their generalization ability across different detectors and often demands complex and expensive re-training. Therefore, cross-detector matching represents a significant gap in this field, a challenge that this work formalizes and addresses for the first time in the literature.

### 2.3. Coplanarity Constraints in Computer Vision Tasks

The utilization of planar surfaces or, alternatively, the coplanarity of points lying on the same plane, is widely adopted to solve computer vision tasks. For example, in 3D scene reconstruction, recent works have demonstrated the benefit of exploiting coplanarity, improving the accuracy of the structure estimation while providing a more reliable data association [21,22,23]. Extensively, the application of these primitives for 3D pose estimation has also emerged with relevant works [24,25,26,27], which highlight planes as a compact representation that allows for efficient and robust pose estimation in large-scale environments. Not only the primitives but also their properties (e.g., homography transformations) are applied, and they are leveraged for tasks such as camera calibration [28,29] and image alignment [30,31], among others.

## 3. Analyzing Keypoint Cross-Detection in Visual Localization

**Figure 2:**
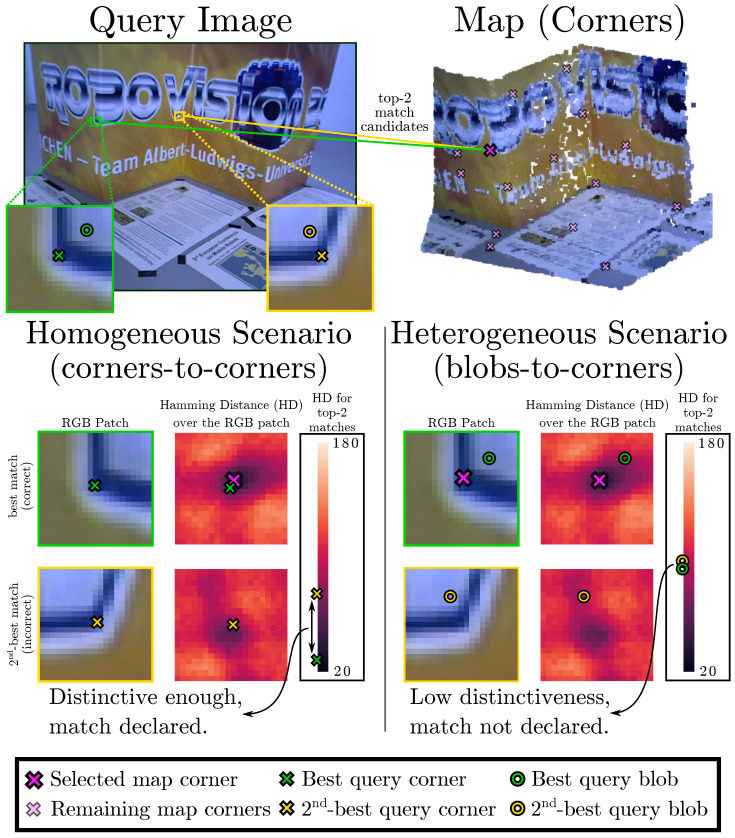
Simple example illustrating the lack of distinctiveness between matches candidates in cross-detector scenarios. The map is created using corner keypoints, so the homogeneous scenario uses corners (×) in the query image, while the heterogeneous employs blobs (○). We depict the zoomed-in RGB patch around the best and the second-best query matches for one map keypoint for both scenarios. The comparison between the Hamming distances between the first and second matches reveals that the best match in the homogeneous case is substantially more distinctive than the heterogeneous case. Consequently, typical feature matching processes relying on this distinctiveness will suffer in the cross-descriptor case.

Performing VL with heterogeneous keypoint detectors poses a major problem, as the keypoints in the query image ($p^{D^{\prime }}$) extracted with a keypoint detector, $D^{\prime }$, represent different physical entities than those keypoints in the map ($P^{D}$) extracted with a keypoint detector, *D*. As illustrated in Figure 2, those keypoints may be proximate, but they are not in the same location. This significantly hinders the traditional feature matching process, making it more difficult to discern the correct correspondences. Moreover, it exacerbates the overall localization problem by adding an extra error term (see Section 3.2) in the cross-detected correspondences.

### 3.1. Cross-Detector Feature Matching

In the scope of camera localization, feature matching methods assume that the same physical entity is represented in both sets of keypoints (query image and map) with almost identical visual descriptors. Thus, to declare a corresponding pair, we expect a query keypoint to have strong descriptor similarity to the first-best candidate in the map while also having enough distance to the second-best one [7]. However, when different detectors are employed, this assumption does not hold.

For a better understanding, we illustrate this problem in Figure 2, for which the evaluation of feature matching is performed for the homogeneous case, where both map and query image employ a corner detector, as well as for the heterogeneous case, where a blob detector is used on the query image. In both scenarios, the matching of a corner feature in the map is evaluated against two candidates in the query image by measuring the difference between the descriptor distances (e.g., Hamming distance) of the first-best and second-best matches. It can be seen how, for the homogeneous case, the distinctiveness between the two candidates is high enough to declare with confidence the correct match. For the heterogeneous case (blob-to-corner), the distance between the best and second-best matches is minimal, making the declaration of the right match unreliable.

### 3.2. Cross-Detector Error

Let M be the set of established correspondences, where each $m_{i}=\left\{p_{i},P_{i}\right\}\in M$ is a match between a keypoint in the query image $p_{i}$ and a 3D keypoint in the map $P_{i}$. Commonly, given a set of matches, VL is formulated as a Least Squares Estimation (LSE) problem:

(1) $$\hat{x}=\underset{x}{\mathrm{argmin}}\sum_{i}{\left(\Pi \left(x\right)\;P_{i}-p_{i}\right)}^{2},$$

where x∈ SE(3) refers to the relative transformation of the camera w.r.t. the map frame and Π(·) is the projection matrix.

**Figure 3:**
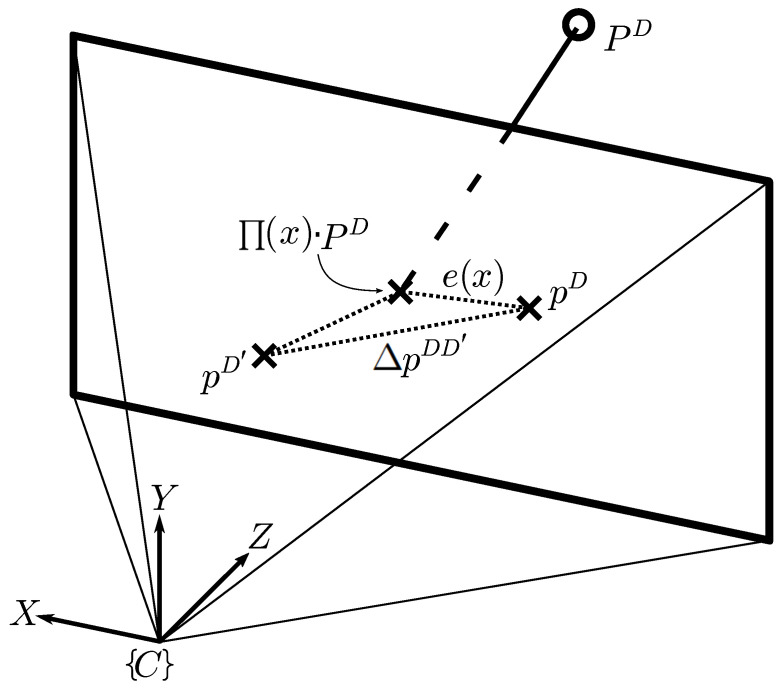
Overview of reprojection error in cross-detector scenario. A 3D map point ($P^{D}$) is projected in the query image, with corresponding keypoints $p^{D}$ and $p^{D^{\prime }}$ for detectors *D* and $D^{\prime }$. Both standard reprojection error (e(x)) and cross-detector error ($\Delta p^{DD^{\prime }}$) are depicted. Errors are exaggerated for clarity.

Particularizing to the cross-detector scenario, the correspondences are pairs between a keypoint ($p^{D^{\prime }}$) in the query image extracted with a keypoint detector, $D^{\prime }$, and a 3D point ($P^{D}$) in the map extracted with a different detector *D* (see Figure 3). Then, Equation (1) can be reformulated as follows:(2)
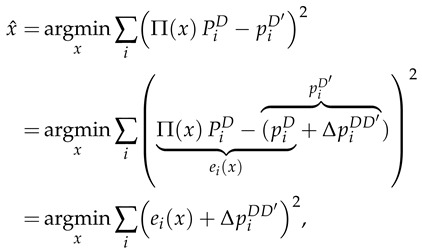

where $e_{i}\left(x\right)$ refers to the standard reprojection error, and $\Delta p^{DD^{\prime }}$ stands for the cross-detector error, representing the error due to the use of different detectors in the feature match.

Provided that $\Delta p^{DD^{\prime }}$ acts as an additive term in the LSE problem, we can argue that the solution of the cross-detector VL will remain the same as in the homogeneous scenario, as long as the cross-detector error distribution is zero-mean [32], i.e., $E[\Delta p^{DD^{\prime }}]=0$, which is experimentally validated in Section 5.

## 4. CoplaMatch: Coplanarity-Constrained Feature Matching

The analysis presented in the previous section stresses the difficulty of declaring reliable matches in cross-detector scenarios. This observation motivates our main contribution: CoplaMatch, a novel approach based on geometric constraints, which allows the establishment of reliable matches in cross-detector scenarios. As discussed in Section 3.1, conventional descriptor similarity approaches face great difficulties because keypoints extracted with different detectors tend to represent different physical entities, subsequently leading to low descriptor distinctiveness. To enhance the matching of cross-detected keypoints, we define the Coplanar Feature Groups (CFGs), which are sets of coplanar keypoints annotated with their respective feature descriptors.

Given the set of CFGs in the map, namely $G_{M}=\{{\mathrm{CFG}}_{j}^{M},\forall j=1,\ldots ,J\}$ and the query set $G_{Q}=\{{\mathrm{CFG}}_{k}^{Q},\forall k=1,\ldots ,K\}$, our goal is to make reliable matches between keypoints from the map (i.e., landmarks) and keypoints from the query image, by forcing them to fulfill a geometric restriction, i.e., features belonging to a ${\mathrm{CFG}}_{k}^{Q}$ must be matched to features of a ${\mathrm{CFG}}_{j}^{M}$. This constraint lies at the core of this work, and it allows us to replace the one-to-one descriptor-based distinctiveness verification of current matching methods with a set-to-set geometric verification, which is applicable to both homogeneous and heterogeneous settings of keypoint detectors. Concretely, our proposal exploits the projective transformation (i.e., homography) that applies between a set of observed coplanar features in the query image and coplanar features in the map. It should be noted that, to establish correspondence pairs of cross-detected keypoints, we assume that the keypoints of both map and query image are described in a common domain, either by using the same feature descriptor or by projecting them to a common space where their similarity can be measured with a given metric, e.g., the Euclidean norm [3].

**Figure 4:**
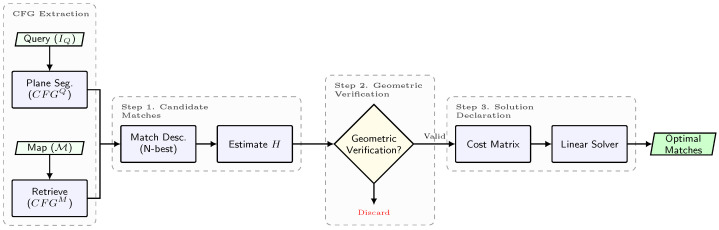
Overview of the CoplaMatch algorithm for cross-detector visual localization. The method takes Coplanar Feature Groups (CFGs) from the query and map as input. It establishes correspondences by relaxing descriptor similarity (Step 1) and enforcing geometric consistency via homography estimation (Step 2). Finally, a linear assignment solver determines the optimal set of matches based on inlier consensus (Step 3).

Imposing coplanarity constraints comes at a price. While annotating the coplanarity information in the map has to be peformed just once in an offline procedure, for the query image, an extra process is required that demands either the depth of the detected keypoints (e.g., from an RGB-D camera) or performing plane segmentation in the RGB image (e.g., using Plane R-CNN [33]), which can be time-consuming. Specifically, we generate CFGs by projecting the segmentation masks onto the image and assigning each keypoint to the plane index of the mask pixel it occupies. This assumes that keypoints falling within a mask belong to that physical plane. While segmentation noise at boundaries can occur, our RANSAC-based homography estimation (Step 1) naturally filters out keypoints that are spatially inconsistent with the dominant plane. Nonetheless, in practice, this time-consuming process is a manageable limitation since, here, we are interested in a feasible solution for global camera localization or for resetting the accumulated odometry drift in a pose-tracking task. In both cases, real-time performance is not strictly demanded, and indeed, the current state-of-the-art SLAM architectures, such as Hydra [34], rely on such segmentation processing. With that being said, the CoplaMatch algorithm can be decomposed into the following three steps (illustrated in Figure 4 and summarized in Algorithm 1).

**Figure 5:**
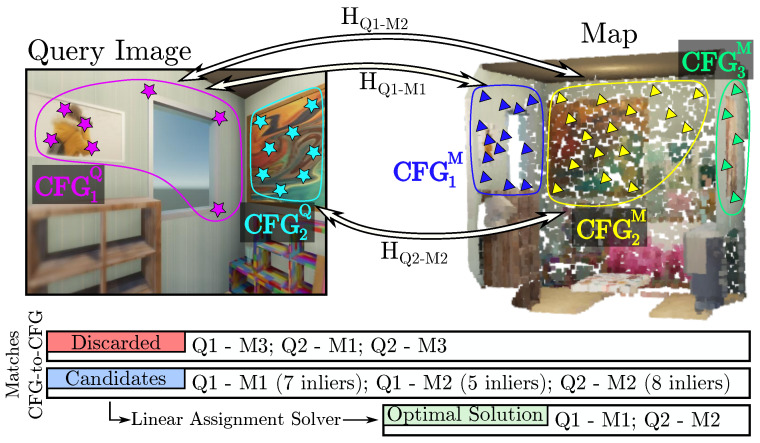
**CoplaMatch** is presented here in a simple example between a query image and a map, but only considering structural planes (i.e., walls) for clarity. Given the CFGs from the query and the map, potential correspondences between them are established using a descriptor-based method. For each <${\mathrm{CFG}}_{k}^{Q}$, ${\mathrm{CFG}}_{j}^{M}\;\;\;>$ pair (referred to as Qk−Mj in the illustration for clarity), if enough feature matches are available, a homography, *H*, is estimated and used to verify geometrically whether the matches are consistent. Finally, the optimal <${\mathrm{CFG}}_{k}^{Q}$, ${\mathrm{CFG}}_{j}^{M}\;\;\;>$ correspondences are selected by solving a linear assignment problem between the candidates. Note that, in the illustration, discarded pairs represent either not enough matches to estimate a homography or that the homography has not passed geometric verification.

**Step 1. Candidate matches between CFGs:** For each ${\mathrm{CFG}}_{k}^{Q}$, we establish putative matches with all ${\mathrm{CFG}}_{j}^{M}\in G_{M}$ (lines 3–5 in Algorithm 1). First, for each pair <${\mathrm{CFG}}_{k}^{Q}$, ${\mathrm{CFG}}_{j}^{M}\;\;\;>$, we establish matches between their features based on an N-best similarity descriptor, without forcing a minimum similarity between them, to accommodate relaxed matches because of cross-detected keypoints. Empirically, we have verified that a value of N=3 is a good trade-off to limit the number of matches and not miss plausible candidates. Then, within a RANSAC framework, we solve for the homography $H_{Qk-Mj}$ with a maximum number of supporting keypoint pairs (inliers). An example is illustrated in Figure 5, where, from a set of 6 possible <${\mathrm{CFG}}_{k}^{Q}$, ${\mathrm{CFG}}_{j}^{M}\;\;\;>$ pairs (K=2 and J=3), only 3 of them (<${\mathrm{CFG}}_{1}^{Q}$, ${\mathrm{CFG}}_{1}^{M}\;\;\;>$, <${\mathrm{CFG}}_{1}^{Q}$, ${\mathrm{CFG}}_{2}^{M}\;\;\;>$ and <${\mathrm{CFG}}_{2}^{Q}$, ${\mathrm{CFG}}_{2}^{M}\;\;\;>$) are considered as correspondence candidates, that is, a homography is established between them. It is worth noting that segmentation errors are naturally handled in this step: over-segmented regions simply result in valid partial matches against the map plane, while under-segmentation (merged planes) is mitigated via this RANSAC process, which rejects features belonging to the non-dominant plane as outliers.
**Algorithm 1:** CoplaMatch feature matching
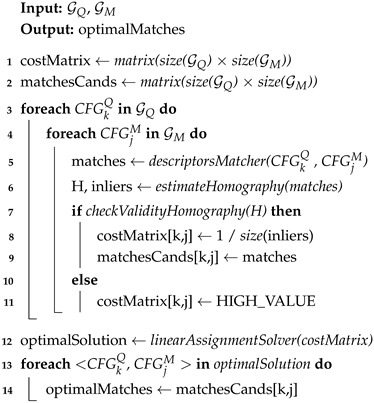



**Step 2. Geometric Verification:** Each obtained candidate CFG match <${\mathrm{CFG}}_{k}^{Q}$, ${\mathrm{CFG}}_{j}^{M}\;\;\;>$ is represented with a homography, $H_{Qk-Mj}$. We can now exploit their R,t decomposition [35] to filter out spatially inconsistent pairs:

- **Rotation Verification.** The estimated homography must be orientation-preserving; that is, the keypoints of any ${\mathrm{CFG}}_{k}^{Q}$ must keep the spatial ordering of their counterparts in ${\mathrm{CFG}}_{j}^{M}$. Otherwise, these CFGs belong to different physical planes. This property is verified through the determinant of the rotational submatrix $H_{R}$ of the estimated homography *H* (2×2 upper-left submatrix):
  (3) $$\det(H_{R})\geq 0.$$
- **Translation Verification.** The R,t decomposition of a homography, *H* [35], gives an up-to-scale translation vector, *t*, between the plane in the map and the camera image plane. Yet, this scale can be approximated from the depth information contained in the reference map, therefore obtaining an estimation of *t*. This step is critical for filtering degenerate homographies caused by the spatial noise inherent to cross-detector keypoints. Since such noise often results in ill-conditioned homographies that yield physically implausible (over-scaled) translations, we set a threshold, $\tau_{t}$, for the maximum acceptable translation.

**Step 3. Solution Declaration:** Notice that, in certain cases, enough keypoints of a <${\mathrm{CFG}}_{k}^{Q}$, ${\mathrm{CFG}}_{j}^{M}\;\;\;>$ pair that do not represent the same physical plane could share similar spatial distribution, leading to an acceptable homography, as illustrated between ${\mathrm{CFG}}_{1}^{Q}$ and ${\mathrm{CFG}}_{2}^{M}$ in Figure 5. In this context, it becomes necessary to solve the data association but while ensuring two requirements: (i) uniqueness, that is, a ${\mathrm{CFG}}_{k}^{Q}$ can only be matched with one ${\mathrm{CFG}}_{j}^{M}$, and (ii) <${\mathrm{CFG}}_{k}^{Q}$, ${\mathrm{CFG}}_{j}^{M}\;\;\;>$ pairs with greater consensus should be prioritized. To this end, linear assignment solvers (e.g., Hungarian or Sinkhorn algorithms [36,37]) are appropriate to find a globally optimal solution over the set of verified <${\mathrm{CFG}}_{k}^{Q}$, ${\mathrm{CFG}}_{j}^{M}\;\;\;>$ correspondences. These solvers require a feasibility score for each pair candidate to quantify the degree of fitness of each candidate in order to select the optimal pairs, taking into account the trade-off between the number of pairs and their fitness. In our case, to promote pairs with high consensus, we set the feasibility score as the number of inliers in the homography estimation, but other alternatives, such as the ratio between inliers and the total number of feature matches, are also applicable. In the example shown in Figure 5, it could be appreciated that, as the pair <${\mathrm{CFG}}_{1}^{Q}$, ${\mathrm{CFG}}_{1}^{M}\;\;\;>$ has a greater consensus than <${\mathrm{CFG}}_{1}^{Q}$, ${\mathrm{CFG}}_{2}^{M}\;\;\;>$, it is prioritized for the optimal solution. As this scoring is computed per CFG pair and depends on geometric consensus, rather than on the total number of detected keypoints, detectors producing denser keypoint sets do not necessarily have an implicit advantage, but CFGs with stronger geometric consensus are naturally favored.

## 5. Experimental Validation

### 5.1. Experimental Setup

This section details the experimental setup employed to evaluate CoplaMatch in cross-detector scenarios. All experiments were conducted on a workstation equipped with an Intel Core i7-4790K CPU and an NVIDIA GeForce RTX 2060 GPU with 6 GB of dedicated memory, ensuring a consistent and robust testbed for performance evaluation.

We performed two distinct sets of experiments. Firstly, a preliminary study was conducted to analyze the cross-detector error (presented in Section 3), in order to validate the suitability of the cross-detected correspondences generated via CoplaMatch for cross-detector VL tasks. Secondly, we assessed the practical applicability and performance of our proposed CoplaMatch within a comprehensive cross-detector VL pipeline, specifically utilizing HLoc [38]. We first built the maps through COLMAP [39] triangulation, using the known camera poses of the mapping sequences and the camera intrinsics. For localization, HLoc was set to use the data from the map available in the top-10 map images retrieved according to NetVLAD [40]. This choice of limiting map images to the top-10 is a common practice in VL to balance localization accuracy with computational efficiency, focusing on the most geometrically coherent and visually similar map views.

A critical aspect of CoplaMatch is its reliance on coplanar feature sets. For all experiments involving CoplaMatch, we exclusively considered feature sets lying on the structural planes (e.g., walls and planar objects attached to them, such as pictures or posters). These structural planes were extracted using Sigma-FP (https://github.com/MAPIRlab/Sigma-FP/tree/plane_segmentation (accessed on 12 November 2025)) [22], a depth-based plane segmentation method. Sigma-FP was selected for its efficiency in processing images (demonstrating lower average processing times compared to RGB-based methods like PlaneRecNet [41]) when depth information is available. The focus on structural planes is motivated by two key factors: it significantly reduces the computational burden by limiting the search space for coplanar groups, and structural planes are inherently robust features in indoor environments [42,43], remaining consistent over time and thus beneficial for long-term SLAM challenges. These identified coplanar groups were then integrated into HLoc to construct planar maps.

To ensure the statistical robustness of our results, our experiments were conducted across sequences from two different real-world datasets, including changes in camera types and environmental conditions: concretely, the *structure_texture* sequences from TUM RGB-D dataset [44], and the Aria Digital Twin dataset [45] using two *Apartment_release* sequences. The Aria Digital Twin dataset is particularly valuable, as it represents a cross-device scenario, where localization is performed using a different camera (front-facing RGB camera) than those used for mapping (grayscale stereo cameras) (https://www.projectaria.com/glasses/ (accessed on 12 November 2025)). The *Apartment_release* sequences are captured in an environment with dynamic changes, further challenging the localization. In this context, our specific reliance on structural planes allows the method to ignore moving objects and anchor to the stable parts of the scene. Both datasets feature distinct trajectories for mapping and localization, allowing for a thorough evaluation of generalization capabilities.

Furthermore, for all experiments, we use five well-known local keypoint detectors, both handcrafted (ORB [9], SIFT [7], FAST [8], and BRISK [10]) and learning-based (SuperPoint [13]), ensuring that our work is not tied to specific algorithm choices and demonstrating consistent performance across heterogeneous setups. This selection provides a representative range of detector types (e.g., corner-based like ORB/FAST/BRISK, blob-based like SIFT, and learned features from SuperPoint), enabling a robust analysis of our method’s performance across different keypoint characteristics. Also, we consider two independent feature descriptors: the handcrafted BRIEF [46] and the learning-based SuperPoint [13], with both being able to describe the five types of keypoints previously mentioned. In this manner, we also evaluate the impact of the feature description on the performance of the matching. Note that, for each experiment, the same feature descriptor is used for the map and the query to ensure the comparability of their descriptors in cross-detector scenarios.

Beyond the general setup, specific algorithmic parameters were carefully chosen and tuned. For instance, in identifying candidate matches, an N-best similarity descriptor with N=3 was empirically selected to balance the number of potential correspondences with the avoidance of missing plausible candidates. Homography estimation relies on a RANSAC framework, employing standard parameters to ensure robust outlier rejection. Furthermore, for geometric verification, a translation threshold $\tau_{t}$ of 5 m was empirically set to filter spatially inconsistent matches derived from degenerate homographies, avoiding the need for specific parameter tuning across different sequences. However, if per-scene tuning is required to maximize robustness, reinforcement learning techniques (e.g., DDPG or TD3) [47] could be employed to automatically adjust these settings based on scene characteristics. Finally, the linear assignment solver’s feasibility score was defined as the number of inliers, promoting solutions with a high geometric consensus.

We compare our proposal with two state-of-the-art feature-matching approaches, with the descriptor similarity matching with Lowe’s criteria [7] and SuperGlue [4]. These comparisons provide a strong baseline against both conventional and advanced matching techniques, highlighting the benefits of our coplanarity-constrained approach in challenging cross-detector environments.

### 5.2. Cross-Detector Error in CoplaMatch Correspondences

As discussed in Section 3, the cross-detector VL problem is equivalent to the standard VL when the expected value of the cross-detector error $\Delta p^{DD^{\prime }}$ is zero. On this premise, it is possible to use common VL techniques based on minimizing the reprojection error to solve cross-detector VL.

In this section, we experimentally analyze the individual impact of the two terms comprising the reprojection error for cross-detected correspondences obtained using CoplaMatch (see Equation (2)): the standard reprojection error e(x), and the cross-detector error $\Delta p^{DD^{\prime }}$. Concretely, we focus on the analysis of the expected value of the errors across a large number of images. By leveraging the linearity property of the expectation, we can decompose the expected value of the overall reprojection error into the sum of the expected values of e(x) and $\Delta p^{DD^{\prime }}$. Consequently, this decomposition allows for a separate examination of both errors.

Given an image pair, we analyze e(x) for correspondences computed using the same detector algorithm between both images. In contrast, we characterize $\Delta p^{DD^{\prime }}$ on individual images, so we can decouple it from the impact of e(x). To do so, we apply different detection algorithms to the image, and then we compute correspondences between the resulting sets of keypoints. For this experiment, we use a large number of image pairs from TUM RGB-D dataset and calculate the correspondences through CoplaMatch. Particularly, we employ ORB as the detector for analyzing e(x); and, to analyze the cross-detector error term, we match ORB keypoints with those obtained with other three different detectors (i.e., SIFT, FAST and BRISK). Note that, to ensure the comparability of different types of keypoints, we processed all with the BRIEF descriptor.

**Figure 6:**
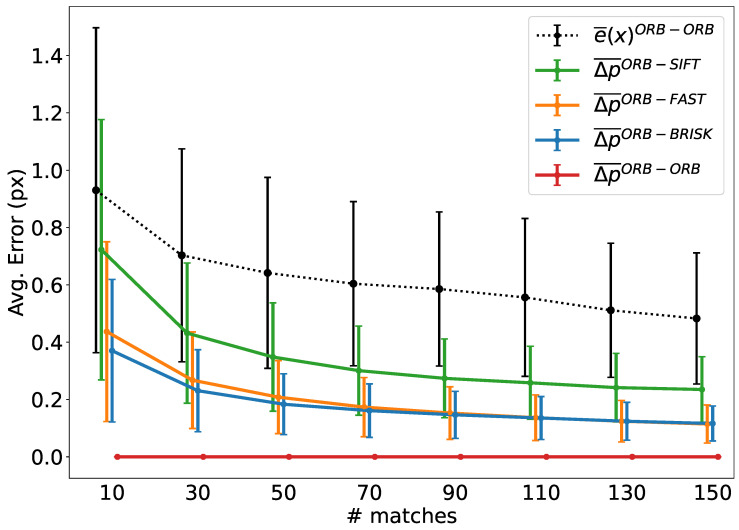
Contrasting standard reprojection error $\bar{e}\left(x\right)$ (for homogeneous scenarios) and cross-detector error ${\bar{\Delta p}}^{DD^{\prime }}$ (in heterogeneous cases) with reference to the number of matches per image pair, with ORB serving as the reference keypoint detector. Note that the total cross-detector reprojection error is the L2-norm of the sum of the two error vectors (see Equation (2)).

Figure 6 compares the average L2-norm of these errors, in terms of the number of matches of the image pairs. It should be noted that to properly characterize and isolate the cross-detector error, only correct matches are selected. The average error is computed as follows:

(4) $${\bar{\Delta p}}^{DD^{\prime }}=\frac{1}{M}\sum_{m=0}^{M}\sqrt{{\left(p_{x,m}^{D}-p_{x,m}^{D^{\prime }}\right)}^{2}+{\left(p_{y,m}^{D}-p_{y,m}^{D^{\prime }}\right)}^{2}}$$

where *M* is the number of correct matches considered, $p^{D}$ states for the keypoints extracted with the detector *D* and $p^{D^{\prime }}$ their correspondences obtained with the detector $D^{\prime }$. It should be noted that the spatial error of the keypoints is separated and computed along the *x*- and *y*-axes of the image.

The results demonstrate that the expected values of both errors, $\bar{e}\left(x\right)$ and ${\bar{\Delta p}}^{DD^{\prime }}$, exhibit larger magnitudes when a limited number of matches (≤30) is considered. However, as the number of matches increases, both errors tend to diminish, suggesting a trend in the reduction in the error with the increasing number of matches. This empirical convergence supports the statistical assumption that the cross-detector spatial discrepancies are uncorrelated and zero-mean, effectively canceling out the cross-terms in the error expansion (Equation (2)). This behavior is visually corroborated by the standard deviation bars in Figure 6, which demonstrate that, while individual cross-detector errors vary, they stochastically cancel each other out when aggregated. We should note that, while the errors decrease simultaneously, they are isolated, and thus, there is no direct interaction between the depicted $\bar{e}\left(x\right)$ and ${\bar{\Delta p}}^{DD^{\prime }}$.

It should be stressed that, in the cross-detector scenario, the total error results from the vector addition of both errors (see Figure 3). As a final note, when the cross-detector errors from individual detectors are compared, it is noticeable that the error for the pair ORB-SIFT, as expected, is greater than the others, given that the natures of the salience points are different (corners vs. blobs).

### 5.3. Analyzing the Computational Cost of CoplaMatch

Generally, introducing constraints incurs an inevitable computational overhead. Particularly, in our proposal, this overhead predominantly comes from the following: (i) plane segmentation within the query image to identify coplanar feature groups, and (ii) applying coplanar constraints during feature matching, which requires the estimation of a homography for each pair of coplanar feature groups between the query image and the map. Thus, it becomes necessary to quantify the additional burden to provide insights into the trade-off between performance and computational cost.

Table 1 presents the average processing times of two plane segmentation methods, PlaneRecNet [41] and Sigma-FP [22]. PlaneRecNet is a learning-based method that identifies planes directly from RGB images. These kind of approaches often estimate depth implicitly, which in turn results in longer processing times. In contrast, depth-based methods such as Sigma-FP depict lower processing times at the cost of requiring depth sensors. It should be noted that the choice of the plane segmentation approach is usually coupled with the available data and the requirements of the application, considering that plane segmentation is performed once per query image. However, it should be noted that, since CoplaMatch is agnostic to the segmentation method, more efficient alternatives can be adopted as they become available. Furthermore, as discussed in Section 4, for the intended applications of global localization and drift reset, the achieved throughput is sufficient.

**Figure 7:**
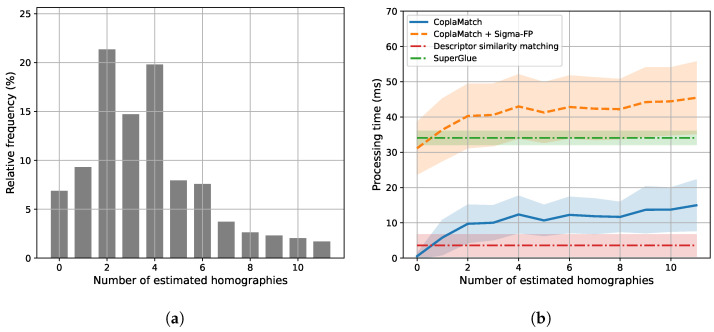
Time analysis based on the number of estimated homographies. (**a**) illustrates the distribution of the number of estimated homographies per image. This histogram illustrates the percentage of images from which a specific number of homographies were estimated.) Meanwhile, (**b**) compares the processing times of the evaluated approaches. (Comparison of processing times for different methods. The *x*-axis indicates the number of homographies estimated, which affects the processing time of CoplaMatch and CoplaMatch + Sigma-FP. The processing times for Descriptor similarity matching and SuperGlue are independent of the number of estimated homographies.)

Once the CFGs of the query are determined, CoplaMatch tries to establish correct correspondences <${\mathrm{CFG}}_{k}^{Q}$, ${\mathrm{CFG}}_{j}^{M}\;\;\;>$. The latter involves the estimation of a homography between the features of each CFG pair, provided that there are sufficient features available in both groups. Figure 7 illustrates the computational time of CoplaMatch relative to the number of estimated homographies between a query image and the map. As expected, the required time exhibits a proportional increase with the number of estimated homographies. However, given the adequacy for concurrent processing of different CFG pairs (lines 3–11 of Algorithm 1), the overall increase in the throughput time is alleviated, although a notable variance is appreciable. In contrast to SuperGlue and the descriptor similarity approach, which exhibit low variance, as they depend solely on the number of matches, CoplaMatch’s variance primarily stems from the variability in the time required to perform RANSAC-based homography estimation. This variability ranges from early convergence, which results in shorter times, to prolonged times in those cases where a solution is not found (i.e., timeout). Additionally, it can be seen that, for >8 homography estimations, the variance increases further, reflecting the parallelization of the algorithm, executed concurrently for this experiment on an eight-core processor. Finally, the histogram shown at the top of Figure 7 indicates that, for ∼81% of query images, the number of homographies to be estimated typically falls between 1 and 6, resulting in an average processing time of ∼13 ms.

In Figure 7, as a reference, we exhibit the computational times for the joint scenario of CoplaMatch with Sigma-FP for plane segmentation. As anticipated, our proposal entails a certain overhead compared to state-of-the-art methods. Yet, this overhead is the cost of guiding the feature matching through the imposition of coplanar constraints to operate effectively in cross-detector scenarios. Although this overhead may limit operations strictly defined as real-time, it operates at ∼20–25 Hz for the Sigma-FP + CoplaMatch case and at ∼10 Hz for PlaneRecNet + CoplaMatch. These computational times are sufficient for tasks such as global localization or for reducing the accumulated odometry drift.

### 5.4. Visual Localization Experiments

We assess the pose recall at different position and orientation thresholds for VL (as in [38]), as well as the median number of matches employed for localization.

**Figure 8:**
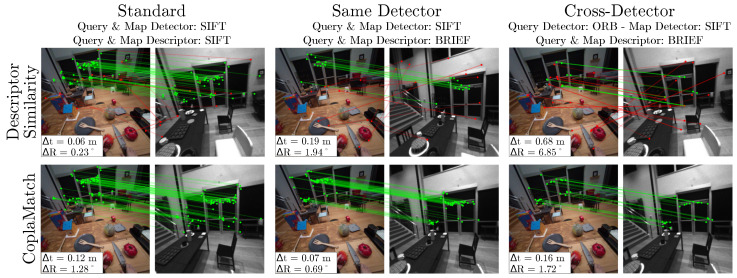
Qualitative results on the Aria Digital Twin dataset comparing feature matching methods for the same query image. For each method, the map image containing the greatest number of correct matches is shown. *Standard VL (**left**)*: Both approaches are able to obtain successful localization. *Same Detector (**center**)*: In this case, as the detector and descriptor use different algorithms, the descriptor similarity approach exhibits certain performance degradation. *Cross-Detector (**right**)*: Finally, for the most challenging scenario, the descriptor similarity approach is not able to establish correct correspondences, whereas CoplaMatch successfully estimates the localization.

For a comprehensive evaluation, we consider two different cases: (i) *Standard*, referring to the classic setting using a feature detector and descriptor from the same algorithm (e.g., ORB detector with ORB descriptor); and (ii) *Cross-Detector*, which represents the scenario where a fixed description algorithm is used but in combination with different keypoint detectors. Table 2 compares both cases for the TUM RGB-D dataset using BRIEF as the fixed descriptor. As expected, we see that the *Standard* is the preferable solution since the description algorithm is designed and optimized for that particular detector, and hence, optimal for setting keypoint matches using their descriptor distance (descriptor similarity matching). However, when moving to the challenging *Cross-Detector* scenario, a significant drop in the number of matches can be observed for descriptor similarity matching (see Figure 8), which has a detrimental impact on the localization performance. The latter is even clearer in the case of an ORB map, where the number of matches is too low and makes localization unfeasible. Additionally, it can be observed that this challenge is more accentuated in blob-to-corner combinations (e.g., SIFT-ORB), which prove more difficult than corner-to-corner (e.g., ORB-BRISK) combinations due to the fundamental difference in feature nature. In contrast, CoplaMatch substantially improves feature matching, increasing the number of pairs and consequently enabling accurate localization with keypoint pairs of a completely different nature. This demonstrates that complementing descriptor similarity with geometry awareness is necessary for cross-detector scenarios.

Table 3 depicts the results for *Cross-Detector* settings. We include the *Same Detector* configuration as a baseline, where the query and map detectors are the same, but unlike the *Standard* setting, in this case, the descriptor algorithm is set to one that can be used with any detector, thus making it different from the detector algorithm employed (e.g., ORB detector with BRIEF descriptor). This experiment is performed using two independent feature descriptors (BRIEF and SuperPoint). The map is created with the detector SIFT, while ORB, BRISK, FAST, and SuperPoint are the detectors used for the query. First, it can be seen that, for the *Same Detector* setting, in which corresponding keypoints refer to the same physical entity, SuperGlue shows impressive performance while both the descriptor similarity matching and CoplaMatch still exhibit competitive results. Yet, in the *Cross-Detector* case, CoplaMatch is the only alternative that presents consistent performance, whereas the performance of the descriptor similarity approach highly depends on the descriptor distinctiveness, and SuperGlue shows particularly poor performance, demonstrating low generalization ability for the cross-detector problem. It should be noted that, as CoplaMatch still relies on descriptors for establishing CFG correspondence candidates, the difference in the median number of correct matches is notable between both descriptors.

Finally, we must highlight that the performance of our proposal is compromised when few planar structures are observed, or they lack textures. This is noticeable in the results of the Aria dataset in Table 3, as well as illustrated in Figure 8, as in this dataset, at times, only one plane is observed and some walls lack texture. This is consistent with the lower number of validated matches seen in Table 3, since the planar regions in Aria are often smoother and yield smaller CFGs than those in TUM RGB-D, directly limiting homography stability and reducing pose recall. In addition, while our experiments on the Aria dataset demonstrate robustness to significant motion, extreme viewpoint changes (e.g., grazing angles) remain a failure case where the underlying descriptor invariance breaks down, rendering geometric verification impossible.

## 6. Conclusions and Discussion

This paper has formally defined and addressed the problem of cross-detector VL, a scenario where distinct keypoint detector algorithms are employed. Such heterogeneity leads to spatial discrepancies among keypoints, reducing descriptor distinctiveness and consequently hindering the typical feature matching based on descriptors’ similarity.

First, we validated, with a wide range of experiments, that the mainstream approach to solving VL, which consists of minimizing the reprojection error, is also applicable to cross-detector scenarios, provided that a significant number of matches are found. In particular, it has been demonstrated that cross-detector correspondences inherently include a cross-detector error, but it acts as an additive term in the LSE formulation, which does not affect the VL solution as long as it exhibits a zero-mean distribution.

Once we validated the feasibility of addressing this problem, we proposed CoplaMatch as the first alternative to find feature correspondences in cross-detector scenarios. To do so, CoplaMatch relaxes the dependence on descriptors’ similarity by imposing geometric coplanarity constraints to guide the matching process. These coplanarity constraints are imposed through 2D homographies between sets of coplanar keypoints, which are denoted as Coplanar Feature Groups (CFGs). In this sense, we perform a geometric verification to discern whether two CFGs (one from the query image and another from the map) represent the same physical plane. This enables the establishment of cross-detector correspondences without heavily relying on descriptors’ similarity. Through a comprehensive set of experiments, we have validated that the correspondences generated via CoplaMatch meet the requirement for the reprojection error minimization approach: the inherent cross-detector error, though present, has a mean of close to zero. This confirmed that, despite the spatial discrepancies introduced via heterogeneous detectors, CoplaMatch effectively produces a set of matches in which the cumulative effect of these errors averages out, thus enabling the accurate performance of cross-detector VL.

However, it is imperative to acknowledge the limitations of our proposal. CoplaMatch fundamentally relies on the observation and detection of planar structures within the scene. Consequently, if the environment lacks discernible structural planes (e.g., outdoors), our method cannot be employed effectively. In such texture-less or non-planar scenarios, the only alternative would be to rely exclusively on descriptor-based matching, which, as shown in our experimental results, suffers from severe performance degradation due to the lack of distinctiveness in cross-detector features. In addition, for extreme scenarios such as observing only parallel planes or those that are similarly textured, the underlying geometry becomes inherently ambiguous, and CoplaMatch may fail to discern the correct solution. Moreover, CoplaMatch introduces an unavoidable computational overhead due to the need for plane segmentation, although such overhead is not excessively large, allowing the operation in the range of approximately 10 to 25 Hz, depending on the plane segmentation technique used.

In conclusion, this work has validated the viability of cross-detector VL, which opens a promising new research direction with a significant impact on the computer vision field. While the method presented in this work serves as a first alternative, there is considerable room for improvement. Future work should explore the cross-detector problem further, investigating alternative approaches that remove the reliance of CoplaMatch on planar observations and enabling the performance of cross-detector VL outdoors or in unstructured environments also. Furthermore, a crucial direction is to extend this work with the cross-descriptor problem to fully enable interoperable visual localization between heterogeneous devices. Additional promising directions include exploiting multi-plane constraints to reinforce geometric consistency and integrating learning-based models for robust CFG identification.

## Figures and Tables

**Table 1:** Average times for plane segmentation of 640×480 images from TUM RGB-D dataset using different methods: PlaneRecNet [41] and Sigma-FP [22].

Method	Average Time (ms)
PlaneRecNet (RGB-based)	90.32±4.31
Sigma-FP (depth-based)	30.57±7.29

**Table 2:** Report on VL results of different methods (descriptor similarity matching [7] and CoplaMatch) in the TUM RGB-D dataset for *Standard* and *Cross-Detector* scenarios. *Standard*: same detector and descriptor algorithm. *Cross-Detector*: BRIEF as a common descriptor but different detectors. The rightmost column shows the median inlier matches for localization. Best results are marked in bold.

Case	Method	Map Detector	Query Detector	% localized Queries			Med. Match. per Query
				0.1 m, 1°	0.25 m, 2°	0.5 m, 5°	
Standard	Descriptor similarity	SIFT	SIFT	**98.28**	**100.00**	**100.00**	**345**
		ORB	ORB	**94.83**	**100.00**	**100.00**	330
	CoplaMatch	SIFT	SIFT	**98.28**	**100.00**	**100.00**	324
		ORB	ORB	87.93	**100.00**	**100.00**	**547**
Cross-Detector (Common Descriptor: BRIEF)	Descriptor similarity	SIFT	SIFT	63.79	82.76	93.10	33
			BRISK	53.45	75.86	91.38	36
			FAST	25.86	39.66	72.41	15
			ORB	39.66	65.52	87.93	56
		ORB	SIFT	0.00	10.34	13.79	11
			BRISK	6.90	18.97	27.59	15
			FAST	5.17	12.07	17.24	7
			ORB	15.52	24.14	29.31	25
	CoplaMatch	SIFT	SIFT	**84.48**	**96.55**	**100.00**	**126**
			BRISK	**81.03**	**98.28**	**100.00**	**130**
			FAST	**56.90**	**79.31**	**98.28**	**88**
			ORB	**58.62**	**89.66**	**100.00**	**135**
		ORB	SIFT	**34.48**	**62.07**	**77.59**	**77**
			BRISK	**55.17**	**77.59**	**87.93**	**105**
			FAST	**50.00**	**68.97**	**74.14**	**80**
			ORB	**51.72**	**68.97**	**86.21**	**139**

**Table 3:** Evaluation of localization performance for different feature-matching methods across two datasets and two common descriptors (BRIEF [46] and SuperPoint [13]). Results are reported for two query configurations: *Same Detector*, where the query uses the same SIFT detector as the map, and *Cross-Detector*, which reports the average performance when the query uses ORB, FAST, BRISK, or SuperPoint detectors against the SIFT map. Localization success is reported at different accuracy thresholds (position, orientation). Best results are marked in bold. Please note that SuperGlue requires specific training for each descriptor, and a BRIEF-compatible version of SuperGlue was not available for evaluation.

Common Descriptor	Query Configuration	Feature Matching Method	TUM RGB-D *fr3/Structure_Texture*				ARIA Digital Twin *Apartment_Release*			
			% Localized Queries			Median Correct Matches/Query	% Localized Queries			Median Correct Matches/Query
			0.1 m, 1°	0.25 m, 2°	0.5 m, 5°		0.1 m, 1°	0.25 m, 2°	0.5 m, 5°	
BRIEF	Same Detector	Descriptor similarity	53.45	65.52	70.69	45	**30.00**	53.33	76.67	64
		SuperGlue	–	–	–	–	–	–	–	–
		CoplaMatch	**89.66**	**96.55**	**100.00**	**163**	26.67	**63.33**	**78.33**	**82**
	Cross-Detector	Descriptor similarity	33.19	55.17	65.08	49	**17.92**	39.17	57.09	42
		SuperGlue	–	–	–	–	–	–	–	–
		CoplaMatch	**59.48**	**90.09**	**98.71**	**165**	14.17	**47.50**	**70.00**	**69**
SuperPoint	Same Detector	Descriptor similarity	87.93	96.55	100.00	74	43.33	75.00	88.33	133
		SuperGlue	**94.83**	**100.00**	**100.00**	**1047**	**58.33**	**86.67**	**93.33**	**455**
		CoplaMatch	93.10	**100.00**	**100.00**	363	53.33	78.33	91.67	205
	Cross-Detector	Descriptor similarity	21.38	69.40	87.07	71	20.00	51.25	76.25	81
		SuperGlue	43.97	50.00	50.00	223	14.59	35.41	47.50	107
		CoplaMatch	**79.74**	**98.27**	**100.00**	**356**	**30.00**	**65.00**	**85.42**	**162**

## Data Availability

All datasets employed in this work are publicly available.
